# Estradiol inhibits vascular endothelial cells pro-inflammatory activation induced by C-reactive protein

**DOI:** 10.1007/s11010-012-1482-9

**Published:** 2012-10-31

**Authors:** Émilie Cossette, Isabelle Cloutier, Kim Tardif, Geneviève DonPierre, Jean-François Tanguay

**Affiliations:** 1 Research Center, Montreal Heart Institute, 5000 Bélanger Street, Montreal, QC H1T 1C8 Canada; 2 Département de Sciences Biomédicales, Faculté de Médecine, Université de Montréal, 2900 Blvd Édouard-Montpetit, Montreal, QC H3T 1J4 Canada

**Keywords:** C-reactive protein, Estradiol, Atherosclerosis, Inflammation, Endothelial cells

## Abstract

In addition of being an important inflammatory biomarker and a risk factor for cardiovascular disease, much evidence indicates that the C-reactive protein (CRP) contributes to the atherosclerosis development process. This plasmatic protein synthesized by hepatocytes in response to inflammation and tissue injury induces pro-inflammatory molecules' expression by endothelial cells (ECs). Previous studies showed that the 17β-estradiol (E2) has beneficial effects on vascular cells by reducing in vitro pro-inflammatory molecules expressions in EC. Therefore, we hypothesize that E2 blocks or reduces CRP-mediated inflammatory responses by modulating endogenous production of CRP in EC and/or activation mechanisms. Using human aortic ECs (HAECs), we first evaluated CRP production by vascular EC and second demonstrated its self-induction. Indeed, recombinant human CRP stimulation induces a fivefold increase of CRP expression. A 1-h pre-treatment of E2 at a physiologic dose (10^−9 ^M) leads to an important decrease of CRP production suggesting a partial blockage of its amplification loop mechanism. Furthermore, in HAEC, E2 reduces the secretion of the most potent agonist of CRP induction, the IL-6, by 21 %. E2 pre-treatment also decreased the expression of pro-inflammatory molecules IL-8, VCAM-1, and ICAM-1 induced by CRP and involved in leukocytes recruitment. In addition, we demonstrated that E2 could restore vascular endothelial growth factor-mediated EC migration response impaired by CRP suggesting another pro-angiogenic property of this hormone. These findings suggest that E2 can interfere with CRP pro-inflammatory effects via activation signals using its rapid, non-genomic pathway that may provide a new mechanism to improve vascular repair.

## Introduction

Cardiovascular disease (CVD) is currently the most important cause of death in developed countries. Atherosclerosis, the underlying cause of most CVDs, is a dynamic and progressive inflammatory disease characterized by lipid plaque formation within the arterial wall and luminal reduction. In fact, accumulating data suggest that the inflammatory process plays a central role in the initiation, progression, and the final steps of this pathology as vulnerable plaque rupture [1, 2]. Many risk factors have been associated with the development of atherosclerosis such as age, hypercholesterolemia, male sex, diabetes, obesity, and hypertension [3–6]. As atherosclerosis represents a process of chronic vascular inflammation, investigations have confirmed inflammatory biomarkers as new risk factors. Various plasmatic inflammatory markers are now considered to identify patients with higher risk of future CVD [7]. However, the C-reactive protein (CRP) has emerged as the most powerful predictor and is an extensively studied systemic marker of inflammation [8]. In fact, among other systemic inflammatory mediators, CRP has been widely accepted as a strong and independent risk factor predicting CVD [9]. As so, elevated baseline concentration of high sensitive CRP (hsCRP) correlates with the risk of future atherosclerotic events [10, 11].

CRP, composed of five identical associated and non-glycosylated 23-kDa subunits, is an acute phase reactant induced during inflammation reaching up to 100- to 1,000-fold its baseline plasma concentration in 24–72 h [12]. Synthesized mainly in the liver by hepatocytes in response to inflammation and tissue injury, it was detected in atherosclerotic lesions and coronary artery walls [13]. More recently, it was demonstrated to be secreted by other cell types such as smooth muscle cells (SMCs), macrophages, and endothelial cells (ECs) [14–16]. Initially considered as an inflammatory biomarker of CVD, evidence now suggests that CRP may also participate in all processes of atherogenesis from endothelial dysfunction to plaque rupture [17]. Indeed, CRP has been implicated in reducing mediators of vasodilatation such as nitric oxide (NO) [18], inducing expression of pro-inflammatory molecules by EC [19, 20] and promoting recruitment of leukocytes to vascular lesions [21]. CRP may contribute to lipid content in forming plaques by aggregating low density-lipoprotein (LDL) molecules which upon excessive uptake by macrophages will favor foam cell development [22]. CRP promotes vascular SMC proliferation and migration [23] while slowing down the reendothelialization process by reducing the vascular endothelial growth factor (VEGF)-mediated migratory response of EC after vascular injury [24]. Therefore, CRP may be an important therapeutic target for the prevention and treatment of atherosclerosis.

Women develop coronary heart diseases on an average of 10 years later than men. This has been attributed, at least in part, to the protective effects of female sex hormones, particularly estrogens [25, 26]. In fact, this effect is lost after menopause when the concentration of 17β-estradiol (E2) is reduced drastically [27]. Several studies have shown that E2 has vasoprotective effects and can modulate inflammatory responses [26, 28]. One of its mechanisms of action in cardiovascular protection consists in improving lipid profile by increasing high density-lipoprotein–cholesterol, while lowering LDL–cholesterol [29]. E2 also promotes arterial vasorelaxation and inhibits platelet aggregation by regulating NO bioavailability [30]. Another important role in vasoprotection is to accelerate reendothelialization and repair after vascular injury. We have shown that intravascular delivery of E2 before stent implantation improves vascular healing with accelerated reendothelialization and inhibition of the inflammatory response, reducing in-stent restenosis [31, 32]. E2 also regulates a variety of anti-inflammatory properties such as reducing vascular expression of chemokines, cytokines, and adhesion molecules, therefore decreasing leukocyte recruitment and accumulation into the vascular wall [33, 34].

However, despite the possible role of CRP in atherogenesis, little is dedicated to the investigation of possible therapeutic strategies to reduce its concentration in the atherosclerotic site. In this study, we hypothesized that E2 blocks or reduces CRP-mediated inflammatory response by modulating endogenous production of CRP in EC and/or activation mechanisms. The current study is the first to underline the capacity of E2 pre-treatment to decrease the CRP autoinduction and triggered ILs-6 and -8 cytokines secretion, key players in the inflammatory process. E2 pre-treatment reduces expression of adhesion molecules, VCAM-1 and ICAM-1, induced by CRP. In addition, we assessed the ability of E2 to counteract impairment of EC migration by CRP. As we suggested, E2 partially restored VEGF-promoted migration of CRP-treated EC.

## Materials and methods

### Reagents

Recombinant human CRP (rhCRP) (Lee Biosolutions, St. Louis, MN, USA) was dialyzed for 24 h using a dialysis slide (Fisher Scientific, Ottawa, CAN) with a cutoff of 10 kDa to remove sodium azide from commercial CRP preparations. In a second step, the CRP was purified using a Detoxigel column (Fisher Scientific) to remove contaminating lipopolysaccharide (LPS), and the absence of endotoxin was confirmed by a limulus amebocyte lysate test (LONZA, Walkersvelle, MD). Water soluble 17β-estradiol and LPS from *Escherichia coli* 0111:B4 were obtained from Sigma Aldrich (St. Louis, USA). Enzyme-linked immunosorbent assay (ELISA) kit OptEIA for the measurement of ILs-6 and -8 are from BD Biosciences (Mississauga, ON, CA). NG-nitro-l-arginine-methyl ester hydrochloride (L-NAME.HCL) was purchased from Enzo Life Sciences (Farmingdale, NY, USA). Recombinant human VEGF_165_ and human VEGF ELISA development kits were provided from Peprotech (Rocky Hill, NJ, USA), while (S)-nitroso-*N*-acetylpenicillamine was from Tocris Bioscience (Ellisville, Missouri, USA). Monoclonal anti-human/mouse/porcine CRP antibody, monoclonal anti-human VEGF receptor-2 (VEGFR-2)/KDR-phycoerythrin (PE), and mouse IgG1 PE isotype control were purchased from R & D systems (Minneapolis, MN, USA). The anti-actin (I-19) antibody, goat anti-mouse IgG–HRP, and donkey anti-goat IgG–HRP were obtained from Santa Cruz Biotechnology (Santa Cruz, CA, USA).

### Cell culture

Human aortic EC (HAEC) were purchased from LONZA and used in experiments at passages 4–6. Cells were cultured with EGM-2MV BulletKit (LONZA) supplemented with 5 % FBS, 0.6 % HEPES, and maintained at 37 °C in a 5 % CO_2_ humidified incubator. All FBS used were pre-treated with 1 % charcoal to eliminate endogenous estrogens. For this, charcoal was added to FBS, gently mixed for 1 h at room temperature before filtration on a 0.22-μm filter and stored at 4 °C. HAEC were plated in six-well plates (Costar, Corning, NY, USA) at 1.5 × 10^4^ cells/cm^2^ and cultured to 80–90 % confluence before being starved in EGM-2MV, 0.1 % FBS for 18 h prior the different treatments.

### Analysis of protein expression by western blot

To evaluate the self-induction of CRP and adhesion molecules (VCAM-1 and ICAM-1) protein expression, HAECs were first treated with rhCRP at different doses (1, 2.5, 5, 10 and 25 μg/ml) for 24 h. To evaluate the impact of estrogen on these inductions, cells were pre-treated with E2 (10^−8^ or 10^−9 ^M) for 1 h before being exposed to rhCRP at 25 μg/ml for 24 h. Single treatments were used as reference controls. After the incubation period, cells were lysed using lysis buffer (20 mM Tris–HCl, 150 mM NaCl, 1.2 % Triton X-100, 1 mM EGTA, 1 mM EDTA, 1 mM PMSF, 15.1 μl/ml aprotinine, 10 μg/ml leupeptin, and 1 mM NaVO_3_). Total protein extract was quantified by Bradford technique (Bio-Rad). Equivalent amount of protein (20 μg) was migrated on a 15 % sodium dodecyl sulfate gel (SDS-PAGE). rhCRP (5 ng) was added to SDS-PAGE as positive control. Proteins were transblotted to a polyvinylidene difluoride membrane that was soaked in 5 % non-fat dry milk prepared in TBS-T [Tris-buffered saline (63 mM Tris–HCl, 7.3 mM NaCl) containing 0.1 % Tween 20] for 1 h at room temperature to block non-specific binding. Membranes were then incubated overnight with one of the following primary antibodies: anti-CRP (1/500), anti-actin I-19 (1/1,000), anti-VCAM-1(1/1,000), and anti-ICAM-1 (1/2,000) antibodies. After three washes in TBS-T at room temperature, membranes were incubated with a horseradish peroxidase-conjugated goat anti-mouse IgG (1/10 000) or donkey anti-goat IgG (1/20 000) or donkey anti-rabbit IgG (1/10 000) for 1 h at room temperature. The blots were washed three times with TBS-T and antigen detection was performed using Immun-Star Western C kit (Bio-Rad). The band intensity was analysed by Quantity One program. Results are expressed in the ratio over actin.

### ELISA assay for ILs-6, -8 and VEGF

Cytokines secretion by HAEC was evaluated after stimulation with rhCRP at 25 μg/ml for 24 h with or without a pre-treatment with E2 (10^−8^ or 10^−9 ^M) for 1 h. After the incubation period, supernatants were harvested and centrifuged to remove cells. ILs-6, -8, and VEGF concentrations in culture media were measured using commercially available ELISA kits. All procedures were performed according to the manufacturer’s instructions. All samples were assessed in triplicate.

### Cellular migration assay

HAEC migration mediated by VEGF was assessed in Transwell cell-culture 96 well plates (Corning) equipped with a gelatine-coated polycarbonate membrane with 5-μM pores. Before the assay, cells were pre-treated in six-well plates with E2 (10^−8^ or 10^−9 ^M) for 1 h, stimulated with rhCRP at 25 μg/ml for 24 h or each treatment alone. L-NAME (10^−4 ^M) was added for 30 min before the treatment of E2 and stimulation of rhCRP or the combination of both. Cells were harvested with trypsin–0.05 % EDTA for 2 min, resuspended in EGM-2MV, 1 % FBS and 5 × 10^4^ cells were added in the upper chamber of the Transwell plate and were migrated for 4 h. EGM-2MV and 1 % FBS alone or with VEGF (20 ng/ml) was added to the lower chamber as chemoattractant. For inhibitors' study, L-NAME (10^−4 ^M) was added to the upper and lower compartment and was present throughout the experiment. After 4 h incubation at 37 °C in the presence of 5 % CO_2_, the cells were fixed in methanol, stained with hematoxylin–eosin dye, and the top side of the insert membrane was scrubbed free of cells with a cotton swab. Membranes were removed using a scalpel and mounted on microscope slides with migrated cell face up. Three evenly spaced fields on each membrane were chosen and pictures were taken using an inverse light microscope (CKX41 of Olympus) equipped with a camera (QIMAGING, QICAM, Olympus) to obtain a computer-digitized image. Counts of migrated cells were performed by a person blinded to treatment by means of ImagePro 6.2 software. Each condition was tested at least in triplicate.

### VEGFR-2 expression using flow cytometer

HAEC were pre-treated with E2 (10^−8^ or 10^−9 ^M) for 1 h, stimulated with rhCRP at 25 μg/ml for 24 h or each treatment alone to evaluate VEGFR-2 expression at the cell surface. Harvested cells were resuspended in PBS–0.5 % BSA and non-specific binding sites were blocked with 5 % of normal mouse serum before 20-min incubation with monoclonal anti-human VEGFR-2/KDR-phycoerythrin (PE, 5 μg/ml) or mouse IgG1 PE isotype control (2.5 μg/ml) for 20 min. Cells were then washed with PBS–0.5 % BSA and resuspended in cytometer buffer. System II software for XL/XLMCL flow cytometer was used for acquisition on an Epics XL coulter cytometer. For each sample, 10,000 cells were analyzed by the program Weasel. Data are expressed as mean fluorescence intensities (MFIs) after background subtraction from the corresponding IgG control.

### Statistical analysis

All data were presented as mean ± SEM. Statistical analyses were performed by one-way analysis of variance for multiple testing followed by Dunnett for ELISA, western blot, adhesion molecules analysis (VCAM-1 and ICAM-1) and EC migration assays, and by Tukey–Kramer multiple comparisons post-test for all other experiments. All statistics were performed by the GraphPad InStat software. Probability values were considered significant at *p* < 0.05.

## Results

### Auto-induction of CRP production by HAECs

**Fig. 1:**
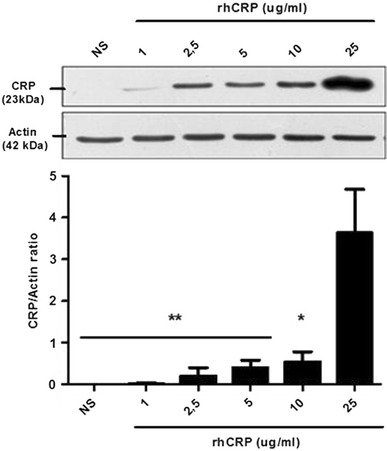
CRP autoinduction in a dose-dependent manner. CRP protein expression was evaluated following HAEC treatment with increasing concentrations of rhCRP for 24 h. CRP protein level was determined by western blot analysis and normalized to the level of β-actin protein. Data represent the mean ± SEM (*N* = 4). **p < 0.001 versus CRP 25 μg/ml; *p < 0.01 versus CRP 25 μg/ml

Self-induced CRP protein expression in vascular EC was investigated. There was no CRP protein detectable under basal (non-stimulated cells) condition. Incubation of HAECs with rhCRP enhanced the CRP protein level in a dose-dependent manner (Fig. 1). A fivefold increase in CRP self-induction was obtained with 25 μg/ml of rhCRP when compared to 10 μg/ml. This increase was significant compared to all other rhCRP concentrations. The dose of 25 μg/ml was selected to conduct further investigation.

### E2 inhibits the auto-induction of CRP by HAECs

**Fig. 2:**
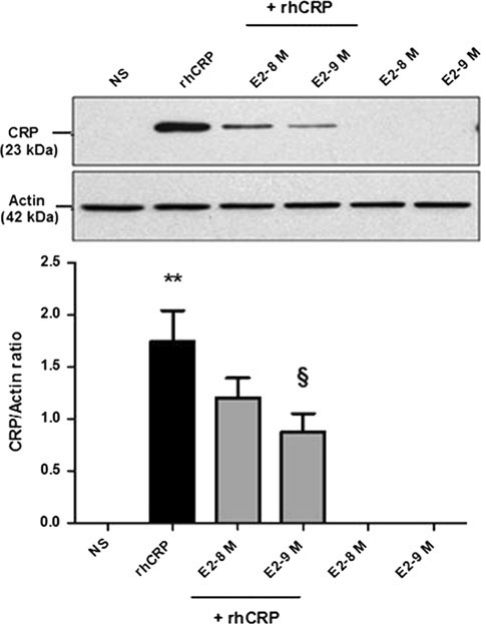
CRP self-induction and negative regulation by E2 pre-treatment. CRP protein expression was evaluated following a 1-h E2 pre-treatment (10^−8^ and 10^−9 ^M) and a 24-h stimulation of rhCRP (25 μg/ml) either alone or in combination. CRP protein level was determined by western blot analysis and normalized to the level of β-actin protein. Data represents the mean ± SEM (*N* = 8). **p < 0.001 versus not stimulated (NS); ^§^ *p* < 0.01 versus CRP

To evaluate the capacity of E2 to reduce or block the CRP self-induction, cells were pre-treated with a supraphysiologic and physiologic doses (10^−8^ and 10^−9 ^M) of E2 before the rhCRP stimulation. HAEC treated with E2 alone were still negative for CRP protein expression (Fig. 2). When added in pre-treatment before the CRP stimulation, E2 significantly inhibits the CRP self-induction with a 50 % reduction at the physiologic dose of E2 in a dose-dependent manner (10^−9 ^M) (*p* < 0.01).

### E2 reduces CRP-induced pro-inflammatory cytokine response

**Fig. 3:**
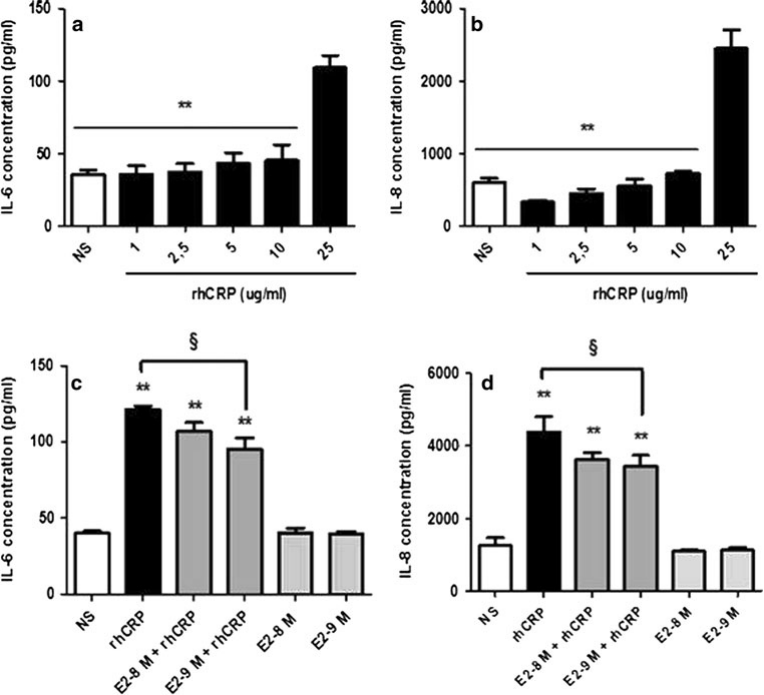
Cytokines (ILs-6 and -8) secreted by HAEC. ILs-6 and -8 secretion was evaluated in the supernatant of HAEC stimulated with increasing concentration of rhCRP for 24 h, a 1-h E2 pre-treatment (10^−8^ and 10^−9 ^M) or both combined. ILs-6 level (**a**, **c**) and -8 level (**b**, **d**) was determined in culture supernatants by ELISA. Data represents the mean ± SEM. *N* = 5 (**a**), *N* = 4 (**b**), *N* = 3 (**c**), *N* = 5 (**d**). **p < 0.001 versus CRP 25 μg/ml (**a**, **b**); **p < 0.001 versus NS; ^§^ *p* < 0.01 versus CRP (**c**). **p < 0.001 versus NS; ^§^ *p* < 0.05 versus CRP (**d**)

As E2 pre-treatment reduces the CRP expression, we have investigated the impact of E2 on IL-6 secretion, one of the most potent agonists of CRP production [12]. In a dose–response study, we first observed a significant increase in IL-6 secretion only with the 25 μg/ml of rhCRP (Fig. 3a). A twofold rise in IL-6 release was observed between the 10 μg/ml CRP dose and the highest dose. An E2 pre-treatment for 1 h at 10^−9 ^M reduced by 21 % the IL-6 secretion triggered by the rhCRP stimulation (Fig. 3c).

We have extended our analysis to another cytokine—the IL-8, an important player of the inflammatory process promoting leukocyte recruitment and adhesion to the endothelium. A response profile similar to the one of IL-6 was observed with a significant increase in IL-8 secretion only with the 25 μg/ml rhCRP stimulation (Fig. 3b). E2 pre-treatment at 10^−8^ and 10^−9 ^M for 1 h significantly reduced by up to 21 % the impact of CRP on IL-8 production (Fig. 3d). E2 by itself, at the tested doses, had no effect on the basal level of ILs-6 and -8 produced by the HAEC.

### E2 reduces CRP-induced adhesion molecule upregulation

**Fig. 4:**
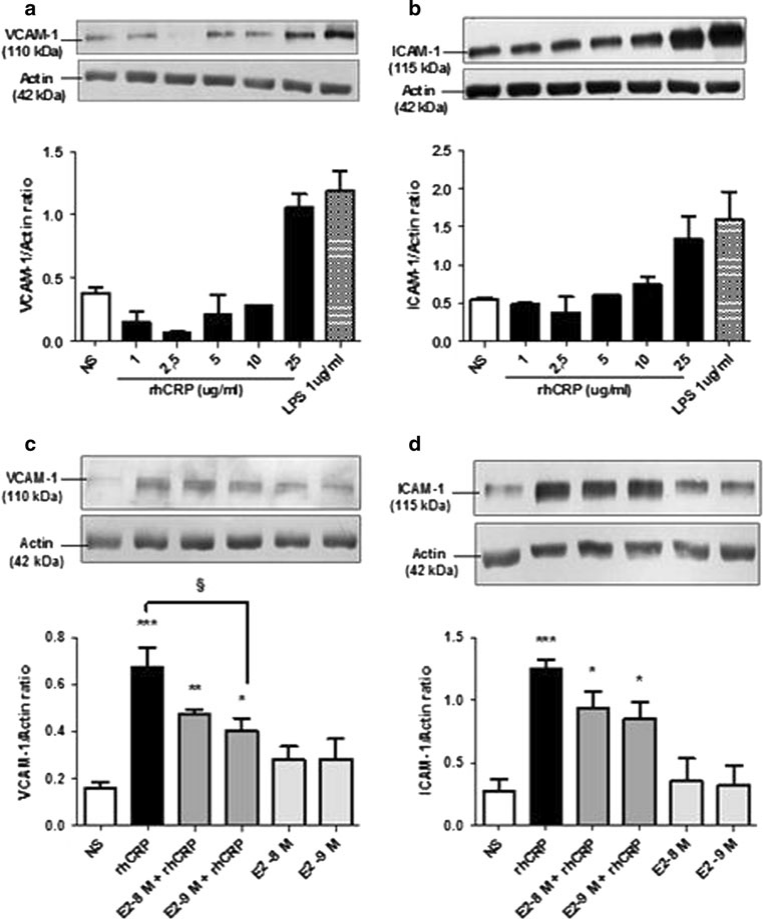
VCAM-1 and ICAM-1 total protein expression in HAEC. VCAM-1 and ICAM-1 protein expression was evaluated in HAEC after stimulation with increasing concentrations of rhCRP for 24 h, a 1-h E2 pre-treatment (10^−8^ and 10^−9 ^M) alone or in combination. VCAM-1 (**a**, **c**) and ICAM-1 (**b**, **d**) protein level was analyzed in cell lysate by western blot and normalized to the level of β-actin protein. Data represents the mean ± SEM. *N* = 2 (**a**), *N* = 2 (**b**), *N* = 4 (**c**), *N* = 4 (**d**). ***p < 0.001, **p < 0.01, *p < 0.05 versus NS; ^§^ *p* < 0.05 (**c**). ***p < 0.001, **p < 0.01 versus NS (**d**)

CRP is known to trigger adhesion molecule expression, such as VCAM-1 and ICAM-1 [19], involved in inflammatory cells' recruitment to endothelial lesion sites. After having observed the capacity of E2 to reduce CRP-induced IL-8 secretion, we evaluated if it could also reduce EC adhesion molecule expression induced by rhCRP. We first evaluated VCAM-1 and ICAM-1 total protein expression in HAECs after stimulation with increasing doses of detoxified rhCRP. Enhanced levels of these proteins were observed only with the highest dose of rhCRP (25 μg/ml) (Fig. 4a, b). This induction was comparable to the one obtained with 1 μg/ml of LPS derived from *E. coli* used as a positive control. Afterward, we investigated VCAM-1 and ICAM-1 protein expression following E2 and rhCRP treatments either alone or in combination. E2 alone had no effect on the expression levels of both adhesion molecules. However, the addition of E2 in pre-treatment (10^−8^ and 10^−9 ^M) before the 24-h rhCRP stimulation decreased by 40 % VCAM (Fig. 4c) and ICAM (Fig. 4d) protein levels with a significant reduction in the case of VCAM (Fig. 4c).

### E2 restores the HAECs' migration reduced by CRP

**Fig. 5:**
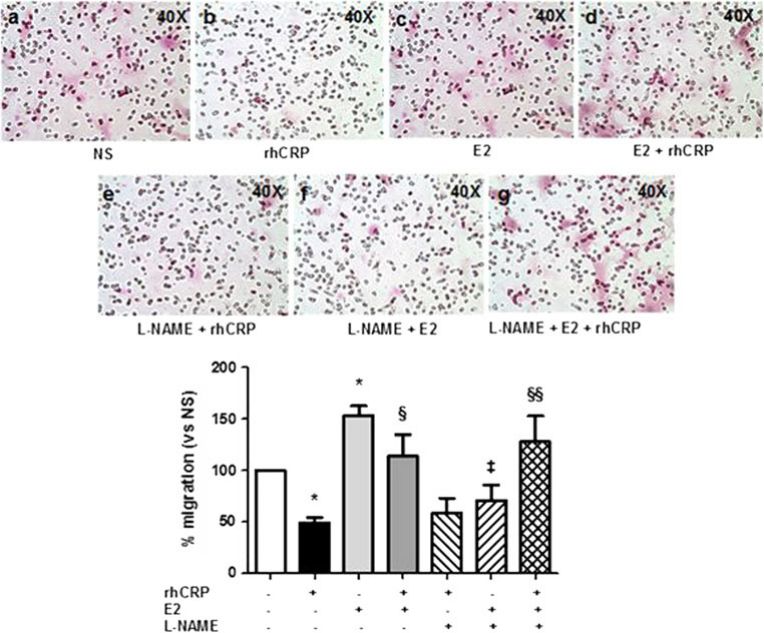
E2 restores the migratory response of CRP-stimulated HAEC to VEGF. HAEC migration toward VEGF (20 ng/ml) was determined in Transwell chamber following E2 1-h pre-treatment (10^−9 ^M) and a 24-h stimulation with rhCRP (25 μg/ml) alone or in combination. L-NAME (10^−4 ^M) was added 30 min before E2 treatment. L-NAME was also added to both the upper and lower compartment in control wells. HAEC migration is shown as a percentage of VEGF-induced increased compared to the average of unstimulated cells in basal condition. Data represents the mean ± SEM (*N* = 3). *p < 0.05 versus NS; ^§^ *p* < 0.05 versus CRP; ^§§^ *p* < 0.01 versus CRP; ^‡^ *p* < 0.01 versus E2

As E2 modulates the pro-inflammatory responses induced by rhCRP and favors an anti-inflammatory pattern, we explored if E2 could improve impaired EC migration by CRP. By means of Transwell migration assays, we first demonstrated that cells stimulated with rhCRP at 25 μg/ml (48.84 ± 4.96 %) for 24 h had a 51 % reduction in their migratory capacity compared to basal condition (Fig. 5, black- vs. white bar). In contrast, cells exposed to E2 at 10^−9 ^M (153.12 ± 9.77 %) for 1 h demonstrated a 49 % increase in migratory activity when compared to cells in media alone (Fig. 5, pale gray- vs. white bar). When added in pre-treatment, E2 (10^−9 ^M) blocks the inhibitory effect of the rhCRP stimulation and restores the basal HAECs' migration response (113.90 ± 21.13 %) to VEGF (Fig. 5, dark gray- vs. dark- vs. white bar).

NO has been reported to be central to the E2-mediated migration and pro-angiogenic activity as well as in the angiogenic response to VEGF [35]. To elucidate if it is through NO induction that E2 restore migration of CRP-treated EC, cells were treated with L-NAME, an inhibitor of NO synthase (NOS) enzyme. A L-NAME (10^−4 ^M) treatment of 30 min was performed before exposure to E2 (70.94 ± 14.65 %) to confirm the implication of NO in E2-mediated pro-migratory effect toward VEGF. A 58 % reduction in HAEC migration was observed compared to E2 treatment alone (Fig. 5, line- vs. pale gray bar). However, after rhCRP stimulation, L-NAME did not prevent the positive effect of E2 on the VEGF-mediated migratory response of CRP-exposed HAEC (127.76 ± 25.03 %) (Fig. 5, scared- vs. dark gray bar). Therefore, these data suggest that NO is not the mechanism by which E2 counteract the inhibition effect of CRP on VEGF-mediated EC migration. The percentage corresponds to the cell count of each condition compared to the average cell count of unstimulated cells.

### Induction of VEGF and VEGFR-2 is not part of E2-mediated migration of CRP-treated EC

**Fig. 6:**
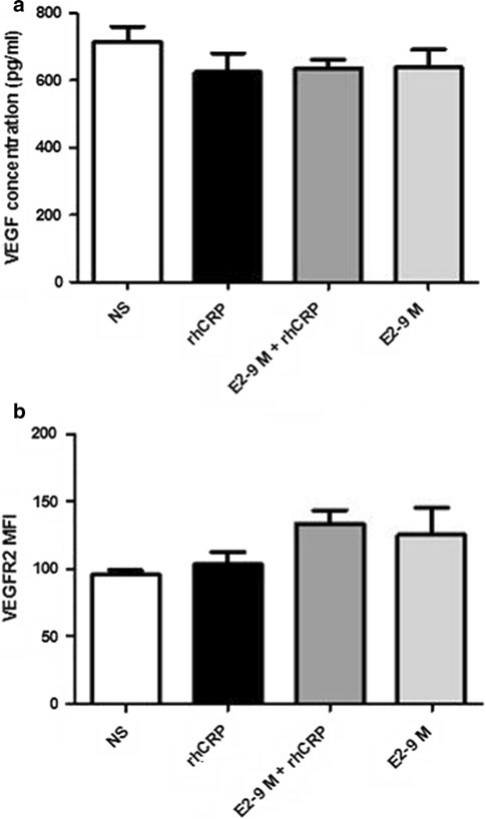
VEGF secretion and VEGFR-2 expression by HAEC. VEGF secretion and VEGFR-2 expression was determined after HAEC's stimulation with a 24 h rhCRP (25 μg/ml) treatment and a 1 h E2 pre-treatment (10^−8^ and 10^−9 ^M) alone or in combination. VEGF was measured by ELISA in culture supernatants of HAEC (**a**). Cell surface VEGFR-2 expression was evaluated by flow cytometry and data are presented as MFI values (**b**). Data represent the mean ± SEM. *N* = 3 (**a**, **b**)

E2 proangiogenic potential is also related to the induction of VEGF production by cells and lead to increased expression of VEGFR-2, an important receptor of the angiogenic mechanism reduces by CRP [36]. To evaluate the involvement of this growth factor in E2-mediated restoration of EC migration altered by CRP, secreted VEGF was measured in cell supernatants by ELISA. Also, VEGFR-2 expression at the cell surface was determined by flow cytometry. However, after treatment with E2 and rhCRP, alone or combined, no significant modulation of both parameters by either E2 or rhCRP was observed (Fig. 6).

## Discussion

It is well accepted that CRP is an important risk factor for CVD playing a critical role in atherogenesis [37–39]. Originally, amplification of CRP production was attributable only to the liver with subsequent deposition of this protein at the inflammatory sites. Its endogenous extrahepatic production by intimal vascular cells was first demonstrated by Yasojima et al. [40]. Then, the cytokine IL-6 was identified as the most important agonist of CRP production that regulates its transcriptional induction in both the HAEC and the liver [16]. Therefore, vascular EC can produce the CRP and the IL-6 contributing to their rapid increased concentration through a retroactive loop at the vascular injured sites [41, 42].

In the present study, purified rhCRP sodium azide and endotoxin free, responsible of reported experimental artefacts [43], was used to evaluate CRP self-induction in vascular EC. A dose dependent increase in self-induced protein expression was observed following stimulation of HAEC with rhCRP. The most significant effect occurred with the highest CRP dose (25 μg/ml), a concentration which could be representative of the exponential augmentation caused by a positive feedback on its own expression. Indeed, a 2.5-fold increase in CRP-stimulating dose from 10 to 25 μg/ml causes a fivefold increase in total CRP level 24 h later, an argument against the possibility that this increase could be simple due to the adhesion and/or uptake of the stimulating protein by cells. Equivalent doses of CRP in plasma represent a very high risk of CVD. However, its concentration in vascular lesion sites can be much greater than in serum. In atherosclerotic plaques, CRP level has been reported to be ten times higher than in a normal artery and seven times superior, as in the liver [40]. Our observations are the first suggesting a CRP synthesis that results from an endogenous autocrine/paracrine loop generated by EC.

The cardioprotective effect of E2 was questioned following the publication of various studies which have tested in vivo hormonal replacement therapy (HRT) [44, 45]. Controversial results showed an increase of hsCRP with the use of HRT in post-menopausal women [46]. Several factors may explain such conflicting results including drug composition (estrogen alone or combined with progesterone), concentration, treatment time, and route of administration. Some of these studies used relatively high concentration of E2, which may result in the development of complications [47]. As so, transdermal E2 did not trigger an increase in plasmatic CRP level compared to the oral formulation, suggesting a first passage effect in the liver [48]. In an animal study, E2 administered subcutaneously to transgenic mice expressing human CRP abolished the overexpression of CRP in the intima following a ligation of a coronary artery. It also reduced vascular inflammation, despite a stable blood level of CRP [49]. Therefore, a targeted delivery of the hormone may play a distinct role and have a beneficial effect for the prevention of atherosclerosis. Our results show that, effectively, a pre-treatment of E2 for 1 h at doses equivalent to its supraphysiologic and physiologic concentration (10^−8^ and 10^−9 ^M) reduces CRP self-induction mechanism dose dependently. These results reveal that E2 attenuates the CRP expression by a rapid non-genomic effect, reflecting another beneficial mechanism of E2 mediated through this pathway.

Having demonstrated that E2 reduced the self-induction of CRP, we confirmed the elevation of IL-6 production by EC with CRP treatment at a dose of 25 μg/ml of rhCRP. Our results are consistent with those of Verma et al. [41] which showed a similar elevation in IL-6 level following addition of a comparable dose of CRP to human saphenous vein ECs. E2 reduces vascular inflammation by altering the expression of adhesion molecules, chemokines, and pro-inflammatory cytokines [26, 33] such as IL-6, in part, through the negative regulation of the transcription factor NF-κB. This factor is also involved in controlling the production of CRP by EC [26, 33, 50, 51]. With the partial inhibition of CRP self-induction and CRP-stimulated IL-6 production, we have shown that E2 could imply a direct inhibition of NF-κB activation and/or an indirect negative effect on IL-6 secretion, interfering with the amplification of CRP production. Likewise, IL-6 acts as a multifunctional cytokine by modulating the hepatic response and participating actively in the inflammatory process of atherogenesis. Indeed, this molecule is produced by multiple cell types present in the atheromatous plaque as EC and is strongly involved in the initiation and maintenance of inflammation, at least in part, by producing CRP [42]. Clinically, the reduction of CRP-induced IL-6 production with E2 pre-treatment shown by our result could indicate a key role of this hormone in the prevention or reduction of cytokine inflammatory response implicated in the development and maintenance of atherosclerosis. An important step in atherosclerosis development is the recruitment of leukocytes in the vascular wall where they become foam cells. This inflammatory stage is favored by an augmentation of chemokine secretion, including IL-8 and MCP-1, and in adhesion molecules expression (ICAM-1, VCAM-1, and E-selectin) at the endothelium [52]. Besides, a substantial enhancement of CRP production in sites of atheromatous lesions further increases expression of pro-inflammatory molecules by EC [19–21]. The important upregulation of ILs-6, -8, ICAM-1, and VCAM-1 expression by HAEC was observed with the 25 μg/ml dose of rhCRP but not the lower dose of 10 μg/ml. This corroborates with the effective dose reported by Devaraj et al. [19, 20]. Another team has demonstrated an ICAM and VCAM induction in human EC with 10 μg/ml CRP and even a lower dose [21], a level of activity that we do not observe with our highly purified rhCRP.

The well-recognized anti-inflammatory activity of E2 could counteract some of the pro-atherogenic activity of the CRP. Indeed, this hormone at 10^−8^ and 10^−9^ M was shown to inhibit leukocyte migration and adhesion by blocking IL-8 secretion in human umbilical vein EC (HUVEC) [53]. E2 was also shown to attenuate monocytes recruitment to HAEC in response to TNF-α [34]. For the first time, we report that an E2 pre-treatment prevents partial HAEC inflammatory response to rhCRP. In fact, it results in a decreased IL-8 secretion and a reduced VCAM-1 and ICAM-1 protein expression, events that could reduce leukocytes recruitment induced by the CRP.

Vascular repair is as important as the reduction of inflammation to prevent progression of atherogenesis process. To allow arterial injury healing, regeneration of a healthy endothelium is essential to restore control of vascular tone, homeostasis as well as anticoagulant, anti-aggregating, and anti-inflammatory properties [54]. Reendothelialization and angiogenesis necessary for endothelium reconstitution involves EC proliferation and migration. However, some evidence suggests that CRP may inhibit these cellular functions [55]. In accordance to these data, a recent article demonstrated that long-term exposure to this plasmatic protein significantly inhibited VEGF-induced migration of HUVEC [24]. In our experimental setting, we observed that a 24-h exposition of HAEC to rhCRP was sufficient to reduce their migration response to VEGF by more than 50 %. On the other hand, E2 has the ability to facilitate vascular repair. Effectively, we previously have demonstrated an improved reendothelialization and vascular healing process after local delivery of E2 at the site of vascular injury [31, 56]. We have also shown the induction of EC proliferation and migration by this hormone via the activation of the estrogen receptor-α and the induction of p38 MAPK and ERK1/2 pathways [57]. In the current study, we demonstrated for the first time that E2 overcomes an important anti-angiogenic effect of the CRP and restores HAEC response to VEGF. A major pro-inflammatory and anti-angiogenic activity of CRP consists to downregulate endothelial NOS expression which decreases NO production by EC [18]. Inversely, E2 is known to promote the production of this important vasodilator associated to its non-genomic activation pathway [30]. NO plays an important role in EC functions including VEGF-mediated migration response [35]. First, we confirmed the role of NO in the pro-migratory effect of E2 using a NOS enzyme inhibitor (L-NAME) and observed a significant reduction of EC migration compared to E2 treatment alone. Interestingly, although this supports NO production as the mediator of E2 effect on EC, the enhanced migratory activity attributed to the E2 pre-treatment of CRP-stimulated EC was insensitive to this NO inhibition. Indeed, using L-NAME, E2 maintained its capacity to counteract the effects of CRP. This suggests that, in the presence of CRP, E2 counteracts the negative effects of this inflammatory protein on EC migration by another mechanism. E2 can inhibit the inflammatory response of CRP by a non-genomic pathway and ERK1/2 may be another cellular signaling target. Indeed, this cellular pathway is known to induce VEGF migration and to be modulated either by CRP and E2 [24, 58]. Therefore, ERK1/2 could be a part of the mechanism for E2 in restoring EC migration impaired by CRP.

To identify the E2 mechanism of action, we investigated the expression of VEGFR-2, a receptor which controls mitogenic action of VEGF. This receptor was reported to be downregulated in EC by CRP. However, in our experimental setting, no difference in the expression of VEGFR-2 or of its ligand was observed following a CRP treatment. Nevertheless, the decrease in VEGFR-2 expression by CRP was only demonstrated at the mRNA level by Yang et al. [36]. On its side, E2 was reported to upregulate VEGFR-2 expression in microvasculature EC after a long-term treatment, showing a more likely involvement of the E2 genomic pathway or a paracrine effect [59]. Indeed, E2 was reported to promote VEGF production which then was responsible to trigger an increased expression of VEGFR-2 and pro-angiogenic activity by a paracrine mechanism [59, 60]. In our experimental setting involving an E2 treatment of 24 h or less of human EC derived from the aortic artery, no induction of VEGF and VEGFR-2 protein level was detected. Altogether, these results point toward a mechanism other than improved NO availability or induced VEGFR-2 expression and VEGF secretion to explain E2 capacity to counteract the effects of CRP on EC.

## Conclusion

Thus, in this study, we demonstrate that EC could express CRP and be a site for CRP self-induction. We illustrate a process of positive feedback production of the protein by vascular cells that could lead to the marked local increase concentration reported in atherosclerotic plaques. Furthermore, the present study highlights a novel vasoprotective role of E2 in the inhibition of this endogenous CRP self-induction, altering its pro-inflammatory activities in vascular EC by a non-genomic pathway. By exploring the angiogenic potential of E2, our study demonstrates for the first time that this hormone restores EC migration altered by CRP. Further investigation will be needed to clarify mechanisms of E2 vascular protection by negative regulation of important proatherogenic inflammatory pathways controlled by CRP.
